# Toxicity of Silver Nanoparticles at the Air-Liquid Interface

**DOI:** 10.1155/2013/328934

**Published:** 2012-12-24

**Authors:** Amara L. Holder, Linsey C. Marr

**Affiliations:** Department of Civil and Environmental Engineering, Virginia Tech, 411 Durham Hall (0246), Blacksburg, VA 24061, USA

## Abstract

Silver nanoparticles are one of the most prevalent nanomaterials in consumer products. Some of these products are likely to be aerosolized, making silver nanoparticles a high priority for inhalation toxicity assessment. To study the inhalation toxicity of silver nanoparticles, we have exposed cultured lung cells to them at the air-liquid interface. Cells were exposed to suspensions of silver or nickel oxide (positive control) nanoparticles at concentrations of 2.6, 6.6, and 13.2 **μ**g cm^−2^ (volume concentrations of 10, 25, and 50 **μ**g ml^−1^) and to 0.7 **μ**g cm^−2^ silver or 2.1 **μ**g cm^−2^ nickel oxide aerosol at the air-liquid interface. Unlike a number of *in vitro* studies employing suspensions of silver nanoparticles, which have shown strong toxic effects, both suspensions and aerosolized nanoparticles caused negligible cytotoxicity and only a mild inflammatory response, in agreement with animal exposures. Additionally, we have developed a novel method using a differential mobility analyzer to select aerosolized nanoparticles of a single diameter to assess the size-dependent toxicity of silver nanoparticles.

## 1. Introduction

As the number of nanotechnology-based consumer products in the marketplace grows, so too does the potential for inhalation exposures to nanomaterials. Aerosolized nanoparticles have been shown to be released during many phases of production: particle synthesis [1–4], handling of dry powders [5] and liquid suspensions of nanoparticles [6], and machining composite materials containing nanoparticles [7]. Experimental studies have shown that engineered nanoparticles released by sprays and powders can potentially deposit in the respiratory system [8–11].

Due to their antibacterial qualities, silver nanoparticles are widely used in consumer products. Nanosilver is present in ~30% of the available products containing nanomaterials [12], and of these, ~14% have a high potential for inhalation exposure [13]. Inhalation exposures are likely to occur with personal hygiene and cleaning products that are intended to be sprayed. Because these consumer products release silver nanoparticles into the breathing zone of consumers, it is imperative to determine the potential hazards associated with inhaling silver nanoparticles.

A safe level for airborne silver nanoparticles has yet to be determined. Inhaled silver has been detected in the blood, liver, brain, and kidneys of exposed rats [14, 15]. Despite the wide distribution of silver throughout the body, no adverse effects were observed in hematology and histopathology assessments at low doses (~0.06 mg m^−3^) [15]. Animals exposed to silver subacutely at a high dose, 3.3 mg m^−3^, showed minimal pulmonary inflammation or cytotoxicity [16]. In contrast, animals exposed to a moderate dose, 0.5 mg m^−3^, showed signs of chronic inflammation in the lungs and abnormalities in the liver [17, 18]. *In vitro* studies with silver nanoparticles have shown stronger effects, with many different cell lines showing reduced viability or oxidative stress response at doses ranging from the order of 1 *μ*g mL^−1^ to 100 *μ*g mL^−1^ [19–21]. Cell studies have also shown a size-dependent effect; the smallest particles (~5–15 nm) required a lower mass dose to cause decreased viability and greater oxidative stress [22–24].

There are several possible explanations for the variation among *in vitro* studies and the differences between the *in vitro* and inhalation studies. Firstly, the properties of the silver nanoparticles used in each study likely differed. The inhalation studies were all performed with metallic silver nanoparticles (10–20 nm) condensed from silver vapor generated from either a spark discharge apparatus [14] or a furnace [25]. Alternatively, all of the *in vitro* studies were performed with silver nanoparticles either synthesized in solution or purchased in powder form, some of which had coatings, and resuspended in aqueous media. Secondly, the exposure route may have affected toxicity. Silver nanoparticles in cell culture media may aggregate into larger particles, obscuring the effects of the nanoparticles, or over time may release silver ions which can also cause a toxic effect apart from that of the nanoparticles [26, 27].

One way to bridge the gap between animal inhalation studies and *in vitro* studies is to expose cells at the air-liquid interface (ALI) [28]. In this method, cells are exposed to air, and aerosolized particles are then deposited directly onto the cell surface. For *in vitro* studies intended to probe particle toxicity associated with inhalation exposure, this approach is thought to be more physiologically realistic compared to exposure in a liquid suspension. This technique has been used to investigate tobacco smoke [29], diesel exhaust [30, 31], smoke from building material combustion [32], flame-generated cerium oxide nanoparticles [33], metal salt nanoparticles [34], and magnetic nanoparticles [35].

Inhalation exposures of engineered nanoparticles have been identified as posing a relatively high risk across the spectrum of potential health and environmental impacts of nanotechnology [36, 37]. An improved understanding of the toxicity of silver nanoparticles is needed because of their widespread use in commercial products, potential for release into the air [12, 13], and evidence of adverse effects in animal inhalation studies [17, 18]. The objective of this work is to evaluate the toxicity of commercially available aerosolized silver nanoparticles on human alveolar epithelial cells exposed at the ALI. Additionally, a novel approach is used to expose cells to particles within a narrow range of diameters, allowing for the first ever measurement of size-dependent toxicity free of the effects of aggregation.

## 2. Methods

### 2.1. Exposure Chamber Design and Characterization

**Figure 1:**
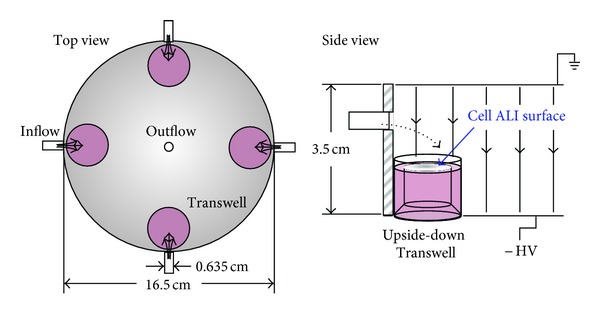
Schematic of the electrostatic precipitator exposure chamber. Aerosol flow entered through four inlets spaced at 90 degrees around the chamber wall and exited through an outlet on the upper plate. Cells were grown on upside-down Transwells that were placed immediately in front of an aerosol inlet.

The exposure chamber consisted of an electrostatic precipitator (ESP) and collagen-coated Transwells (Corning, 12 mm inserts, 0.4 *μ*m pore size, 1.12 cm^2^ growth surface), which contained the cells. The objectives of the chamber design were to (1) direct particles to the cell surface using an electrostatic field, (2) direct air flow across the top of the Transwells rather than directly at the cell surface, and (3) allow for multiple wells to be exposed at once. A schematic of the chamber is shown in Figure 1.

The chamber is constructed of two aluminum plates (15.2 cm in diameter, 6.4 cm thick) forming the top and bottom surfaces and an acrylic pipe (14.6 cm in diameter, 3.5 cm in height) forming the cylindrical wall. Four equally spaced inlets around the acrylic cylinder allow four wells to be exposed simultaneously. The inlet air flows over the Transwells and exits through an outlet in the center of the top plate. An electric field is generated in the chamber by connecting the lower plate to a negative high-voltage DC supply (EMCO, model 4120N) and the upper plate to ground. The clear acrylic wall insulates the ground electrode from the high-voltage electrode and also allows visualization of the wells during an exposure. The Transwells are placed upside down, and cells are grown on what is now the top side of the Teflon membrane (typically the bottom side), in order to minimize the vertical distance that particles must travel before depositing on the cell surface. This orientation maximizes deposition efficiency.

Particle deposition on the Teflon membrane (i.e., the Transwell cell culture surface) was measured with a fluorescein aerosol of a single diameter. The aerosol generation and single-diameter exposure are described below. A foil substrate was placed on the membrane to collect deposited fluorescein particles. Fluorescein was extracted with 0.5 mL of nanopure water, and fluorescence was measured on a plate reader (Molecular Devices, SpectraMax M2). Approximately 100% of the deposited fluorescein can be recovered with this method. The deposition efficiency was calculated as the percentage of mass depositing on the Transwell relative to the total mass entering the inlet, which was derived from measurements of particle number concentration by a condensation particle counter (CPC, TSI model 3025A). The deposition efficiency for each particle diameter (50, 75, and 100 nm) was measured in three wells in three separate experiments, except for 50 nm, which was measured in four separate experiments. In exposure experiments, the dose of nanoparticles depositing on the cells was calculated by applying the deposition efficiency to the inlet aerosol concentration. Although the nanoparticles tested have higher densities than the fluorescein particles, the deposition efficiencies are not affected. Particle motion in the vertical direction is dominated by the balance between the electrostatic force and the drag force; the inertia of the particle is negligible compared to these two forces.

### 2.2. Aerosol Generation and Characterization

Silver (30–50 nm coated with polyvinyl pyrrolidone, PVP 0.2% wt) and nickel oxide (10–20 nm), as a positive control, nanoparticles were purchased from a commercial supplier (NanoAmor, Houston, TX, USA). Nanoparticle stock suspensions were prepared by dispersing the particles in sterile nanopure water with a probe sonicator (Misonix, 3000) at a concentration of 0.5 mg mL^−1^. Suspensions were sonicated on ice at approximately 50 W for 5 min alternating with a 5 min rest on ice. The process was repeated three times to optimize between maximizing breakup of the aggregates and minimizing volume loss to evaporation. The resulting size distribution in suspension was measured by dynamic light scattering (DLS, Malvern Zetasizer Nano). A drop of the suspension was dried on a transmission electron microscope (TEM) grid, and samples were then analyzed with a TEM (Philips EM420). Elemental analysis was performed with a scanning electron microscope (FEI Quanta 600 FEG) equipped with an energy dispersive X-ray spectrometer (EDX, Bruker Quantax 400).

Aerosols were generated with a constant output atomizer (TSI, model 3076), which was cleaned with aqua regia between runs. The nanoparticle aerosols were dried with a diffusion dryer, charge neutralized with a Kr85 source (TSI, model 3012), and mixed with CO_2_ to a concentration of 5%. The size distribution was measured with a scanning mobility particle sizer consisting of a differential mobility analyzer (TSI, model 3081) and the CPC. Aerosol samples for electron microscopy were collected by placing a TEM grid on a Transwell inside the ESP.

### 2.3. Cell Culture and Assays

All experiments were performed with a human alveolar cell line (A549, Sigma ECACC). This cell line has frequently been used to assess the toxicity of nanoparticle suspensions because it is representative of Type II pneumocytes and is a model for the alveolar epithelium [38, 39]. This region of the lung is particularly susceptible to the effects of nanoparticles because it has the largest deposition fraction for particles in the 10–100 nm size range and does not have the protective mucus lining found in the nasal and bronchial regions [40]. Cells were grown in F12 medium using Kaighn's modification (F12K, Invitrogen) with 10% fetal bovine serum (FBS, Invitrogen) and 1% antibiotic/antimycotic (Invitrogen). Nickel oxide nanoparticles were used as a positive control as they have previously been shown to generate more reactive oxygen species compared to other nanoparticles and cause a cytotoxic response in the A549 cell line [38].

Measurements of cellular response were made with several assays commonly used to assess response from nanoparticles [24, 38, 41]. Cytotoxicity was assessed with a lactate dehydrogenase (LDH) assay (kit from Sigma) and methylthiazol tetrazolium (MTT) assay (Sigma). Leakage of the LDH protein is measured as an indicator of a loss of membrane integrity. The exposure medium was collected after nanoparticle exposure and centrifuged for 10 min at 10,000 rpm to remove the nanoparticles from the medium. The extracellular LDH concentration in the supernatant was measured following the manufacturer's protocol. The metabolic activity of the cells was measured with an MTT assay. After the postexposure incubation period, cells were incubated another 1.5 hr with MTT (1 mM) in F12K medium. After incubation, the medium was aspirated, the formazan was solubilized with dimethyl sulfoxide, and the absorbance at 540 nm was measured on a plate reader (SpectraMax M2). A proinflammatory response was assessed by measuring the secretion of the pro-inflammatory cytokine interleukin 8 (IL-8) with an ELISA assay (kit from Invitrogen). IL-8 secretion is routinely measured to assess inflammatory response to aerosols [41]. The exposure medium was collected and centrifuged for 10 min at 10,000 rpm to remove the nanoparticles. The supernatant was kept frozen at −8°C until the assay was performed according to manufacturer's instructions.

### 2.4. ALI Exposure

Cells were plated on collagen-coated Transwell inserts at a density of 10^5^ # cm^−2^ following a protocol modified from Gohla et al. [42]. Briefly, inserts were turned upside down, and 0.15 mL of cell suspension was placed on the bottom of the insert. The insert was placed inside an incubator at 37°C with 5% CO_2_ for 3 hr while the cells attached to the Teflon membrane. The excess medium was removed, and the inserts were placed with the right side up in a 12-well plate and grown submerged (1.0 mL medium in the bottom chamber, 0.5 mL medium in the upper chamber) for two days before the exposure.

In preparation for an ALI exposure, the Transwell inserts were placed upside down inside sterile glass wells (2.6 cm in diameter, 2.2 cm deep), 8 mL of medium was added to the well, and 0.1 mL of medium was placed on top of the insert to prevent the insert from drying out. The glass wells and inserts were then placed inside the chamber for the duration of the aerosol exposure (i.e., dosing period). A second group of wells was placed in an identical chamber to serve as the control group. Each chamber was wiped down with ethanol before the exposure to maintain sterility.

Two ALI exposure scenarios were used in this study: whole aerosol (polydisperse) exposure and single-diameter (monodisperse) exposure. The whole aerosol was drawn into the ESP chamber with the voltage set at −2.4 kV. In this arrangement, a neutral charge distribution with both positive and negative charges was established by passing the aerosol through the Kr85 source; only the positively charged particles deposited on the exposed wells. For the single-diameter exposure, the nanoparticle aerosol was first routed through the differential mobility analyzer to select positively charged particles of a single diameter. This monodisperse aerosol was then drawn into the exposure chamber (kept at −2.4 kV), where the particles deposited on the wells. For both exposure scenarios, a control experiment with no particles, attained by placing a filter (Pall, Fiberfilm T60A20) upstream of the chamber, was conducted simultaneously.

The cells were dosed at the ALI for 2 hr with the whole aerosol or 3 hr with a single-diameter aerosol; the extra hour was intended to increase the mass deposited. After being dosed, the inserts were returned to a 12-well plate, where they were incubated submerged in 1.0 mL of F12K media with 10% FBS at 37°C with 5% CO_2_ for 24 hr. The media was then collected to measure LDH and IL-8 concentrations, and the MTT assay was begun. Each ALI exposure condition was done once on triplicate wells.

### 2.5. Suspension Exposure

For comparison with the ALI technique, cells were also exposed to nanoparticles in liquid suspensions. Cells were plated in 12-well plates at a density of 10^5^ # cm^−2^ and grown for two days before an exposure. Nanoparticle stock suspensions were generated the previous day in sterile nanopure water and diluted with F12K medium with 10% FBS to 10, 25, and 50 *μ*g mL^−1^ immediately before the exposure. We estimate that all particles in suspension deposited in approximately 5 hrs, which results in a deposited dose of 2.6, 6.6, and 13.2 *μ*g cm^−2^ on the cell layer. This dose range was selected to cover the expected ALI concentration and to be comparable to concentration ranges used in similar *in vitro* studies with silver nanoparticles [24, 43]. Cells were dosed with the nanoparticle suspension (1 mL per well) and kept in an incubator at 37°C with 5% CO_2_ for 24 hr. As was done with the ALI exposures, a single dose (rather than repeated dosing) and the 24 hr incubation period were selected to be comparable to previous studies with silver nanoparticles [24, 43]. After the exposure, the medium was collected for the LDH assay and for IL-8 measurement, and the MTT assay was begun. Each suspension exposure condition was done once on triplicate wells.

### 2.6. Statistical Analysis

Results are presented as the median and the 25th and 75th percentiles. Errors were propagated through the calculated parameters using a bootstrap analysis. Significance was assessed between exposed and control wells using a Kruskal-Wallis test. Differences between conditions were deemed significant for *P* values less than 0.05.

## 3. Results

### 3.1. Particle Deposition

Fluorescein particle deposition on the Teflon membrane was measured for three diameters of particles (50, 75, and 100 nm), as shown in Table 1. The deposition efficiency was highest for the larger 100 and 75 nm diameter particles and dropped off for the 50 nm particles. Over time, charge buildup on the chamber wall tended to reduce the deposition efficiency for the smaller-diameter particles. To prevent this, we wiped down the chamber with nanopure water to remove charged particles that accumulated on the chamber surface. Deposition efficiencies varied by <30% between runs and 15–25% between wells at different locations in the chamber in a single run.

### 3.2. Nanoparticle Aerosol

**Figure 2:**
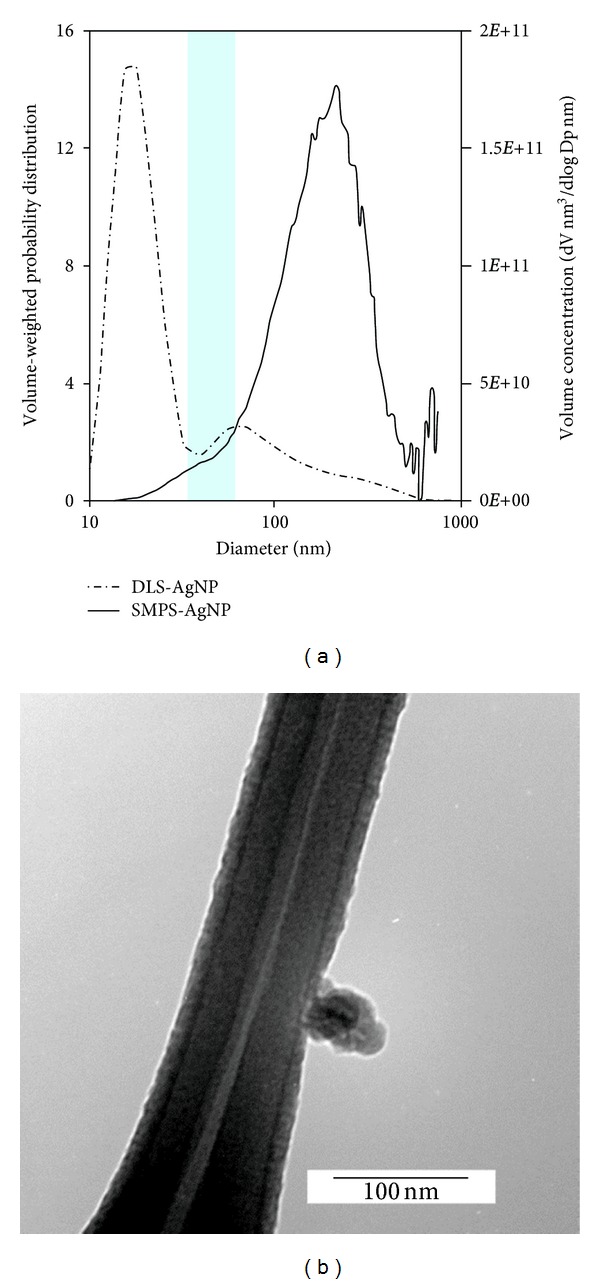
(a) Volume-weighted size distributions of silver nanoparticles in suspension (DLS) and as aerosols (SMPS) (left). The DLS measurement corresponds to the left axis, and the SMPS measurement corresponds to the right axis. The shaded area is the manufacturer's specified range of particle diameters. (b) Transmission electron microscope image of a ~50 nm silver nanoparticle on a lacey carbon grid (right).

Atomizing the suspension of silver nanoparticles resulted in an aerosol that consisted of particles with a geometric mean diameter of 37 nm and a volume-weighted geometric mean diameter of 169 nm (Figure 2). Electron microscopy confirmed that the aerosol particles had the same physical characteristics as the silver nanoparticles in suspension. The particles were approximately spherical with diameters of ~50 nm and were composed of silver with a crystalline diffraction pattern.

A comparison between the volume-weighted size distribution of a silver nanoparticle suspension and aerosol is shown in Figure 2. The volume distribution in suspension was dominated by small particles and peaked at approximately 20 nm with a second mode at 68 nm. The larger mode from the suspension approximately corresponds to the aerosol distribution; an exact match is not expected due to different sizing methods. The smaller size mode was not apparent in the aerosol distribution. This mode was likely composed of PVP released from the particle surface during sonication, as no silver particles in this size range were observed under TEM. Similarly, Foldbjerg et al. [20] attributed a peak at 11 nm after sonication of the same NanoAmor PVP-coated silver nanoparticles to free particles composed of PVP.

### 3.3. Cellular Response to Nanoparticles in Suspension

Exposure to suspensions of nanoparticles was used to gauge the range of responses of this cell line to the silver and nickel oxide nanoparticles. Results of three different assays are presented in Table 2 as a percent of the control group for comparison of different types of exposure. Silver nanoparticle suspensions caused a mild cytotoxic and proinflammatory response. Cell metabolism as measured by the MTT assay decreased with increasing dose of silver nanoparticles. The LDH release in cells exposed to silver nanoparticle suspensions was slightly less than the control value, suggesting that the silver nanoparticles may have interfered with the assay. The nickel oxide suspensions, used as a positive control, also showed a mild dose-dependent cytotoxic response. However, the nickel oxide nanoparticles did not cause a pro-inflammatory response and were actually shown to decrease the release of IL-8 or cause an anti-inflammatory response.

Particles may interfere with cellular assays and cause false toxic or false nontoxic responses to be measured [44]. To check for possible interference of nanoparticles with assay results, we also performed each assay with a known quantity of nanoparticles but without cells. After the incubation period, the nanoparticles were removed by centrifugation (10 min at 10,000 rpm). Neither nanoparticle type affected the MTT assay. However, the silver nanoparticles were found to inactivate or bind LDH protein and thus prevent its measurement; similarly, Han et al. [45] observed that silver nanoparticles in a carbon matrix inactivated LDH protein in a dose-dependent manner. Silver nanoparticles at a concentration of 10 *μ*g mL^−1^ reduced the measurable LDH to 42% of the original concentration, and higher silver nanoparticle concentrations resulted in a greater percentage of the original LDH being bound. Because of this dose-dependent removal, the LDH assay for cells exposed to silver nanoparticles was considered suspect, although at the low doses applied at the ALI the LDH assay may not be strongly affected by the silver nanoparticles. The nickel oxide nanoparticles did not bind the LDH protein at any concentration tested. Similar measurements with IL-8 were performed, and neither the silver nor the nickel oxide nanoparticles bound sizable amounts of the IL-8 molecule. Only about 8% of the IL-8 concentration was adsorbed at the highest nanoparticle concentration of 100 *μ*g mL^−1^.

The dose at the ALI was slightly lower than the lowest dose in suspension when normalized by the cell growth area. The silver aerosol caused a mild cytotoxic effect observed by increased LDH release. Conversely the metabolic rate (MTT) for cells exposed to silver nanoparticles was increased. The silver aerosol also resulted in increased IL-8 secretion. In all cases, the interquartile range was relatively high and none of the observed effects were statistically significant compared to the control. In contrast, the nickel oxide aerosol caused a strong cytotoxic effect with reduction of cellular metabolism (MTT) and membrane integrity (LDH). Similar to the suspension exposure, the aerosolized nickel oxide nanoparticles caused a decrease in IL-8 secretion compared to the control group.

### 3.4. Cellular Response by Size

Cells were exposed to particles of a single-diameter aerosol for 3 hr to achieve doses in the range of 5 to 26 ng cm^−2^ (Table 3). The number dose was calculated from the deposition efficiency measured for each particle diameter, and the surface area and volume dose were calculated from the number dose assuming spherical particle geometry. The dose for each size nanoparticle was different due to the nonuniform size distribution (Figure 2) and the particle charging efficiency varied with size before selection by the DMA. In terms of particle number, the dose was greatest for the 50 nm particles, followed by the 75 nm particles, and then the 100 nm particles. In terms of mass and surface area, doses were greatest for the 100 nm particles and decreased with decreasing diameter. Despite the large variation in the number of 50 nm particles deposited, the mass was not greatly affected, as these particles have very little mass. The decreasing number dose and increasing mass and surface area dose with particle diameter provide an opportunity to investigate the most appropriate dose metric.

**Figure 3:**
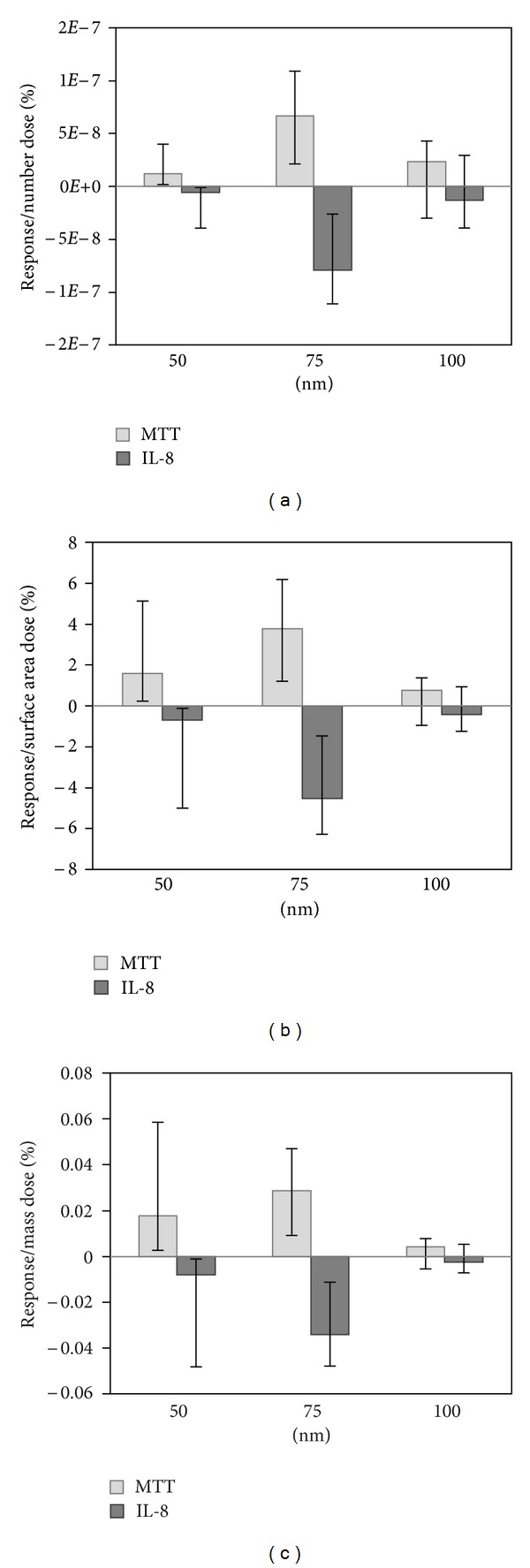
Percent response normalized by (a) number, (b) surface area, and (c) mass dose. Percent response for MTT is calculated as 100%-percent control and for IL-8 as percent control-100% so that an adverse response from each assay is plotted as a positive value and a beneficial response is plotted as a negative value, with zero being no change from the control value. Median values are presented with error bars representing the 25th and 75th percentiles of three replicates for the control and each diameter exposure, except for the 50 nm exposure, for which only two valid replicates were obtained.

To facilitate visualization of the results in Figure 3, the response for single-diameter exposures is compared to the control, and then the difference from 100% is calculated such that an adverse response is positive (i.e., 100%-percent control for MTT and percent control-100% for IL-8). The response is then normalized by number, surface area, and mass dose to facilitate comparison between exposures to particles of different diameters with different dose metrics. Silver nanoparticles of all diameters tested caused a cytotoxic response, as measured by the MTT assay. The response normalized by number dose was greatest for the 75 nm particles and least for the 50 nm and 100 nm diameter particles. In other words, the same number of 75 nm diameter particles caused greater response than either the 50 nm or 100 nm diameter particles. The 100 nm diameter particles caused the lowest response for the mass and surface area dose metric, suggesting that there may be a size threshold for the response to silver nanoparticles. None of the particles caused an inflammatory response that was statistically different from that of the control group.

## 4. Discussion

### 4.1. Toxicity of Silver Nanoparticles

Characterizing the hazard associated with inhaling silver nanoparticles is urgently needed because of their widespread prevalence in consumer products and the high likelihood of their aerosolization during product use. The American Conference of Governmental Industrial Hygienists (ACGIH) has set a threshold limit value of 0.01 mg m^−3^ for soluble silver and 0.1 mg m^−3^ for insoluble silver. These values were determined from epidemiology studies on workers exposed to silver dust, where few adverse health effects were observed apart from the development of argyria [46]. Likewise, rat inhalation exposure studies found no significant effects below 0.1 mg m^−3^ [15]. In the current study, silver nanoparticles in suspensions showed minimal cytotoxicity and only at a high dose of 50 *μ*g mL^−1^ (13.2 *μ*g cm^−2^). Additionally, when exposed at the ALI, cells exhibited no significant toxicity to any dose (from 0.005 to 0.7 *μ*g cm^−2^) of silver nanoparticles of any size. These doses are well above the maximum estimated alveolar dose of 0.001 *μ*g cm^−2^ for a worker breathing at a rate of 1 m^3^ hr^−1^ at the ACGIH recommended threshold limit value for silver of 0.1 mg m^−3^, assuming a fraction depositing in the alveolar region of 0.3 and an alveolar surface area of 75 m^2^. The ALI dose is also well above the estimated dose from exposure to consumer products containing silver nanoparticles. Quadros and Marr [8] estimated a dose of 75 ng of silver from the worst case exposure to consumer products containing nanomaterials, resulting in an alveolar dose of 0.015 pg cm^−2^, seven orders of magnitude higher than the dose at the ALI. Our results suggest, in agreement with the ACGIH threshold limit value, that a onetime exposure to silver nanoparticles from consumer products or in the workplace will not cause adverse effects. We recommend future studies with the ALI system incorporating repeated exposures, which are more likely to occur than the single acute dosing that we have used here and which are common in conventional toxicity testing.

These results fall within the range of values reported in the literature for *in vitro* assessments. Measurements of cytotoxicity of silver nanoparticles in mammalian cells have shown large variability, with concentrations causing 50% viability reductions (lethal dose 50, LD_50_) ranging from 0.8 *μ*g mL^−1^ in media [47] to 1 mg mL^−1^ [48]. Some of the variabilities in these results may be due to the different susceptibility of different cell types to silver nanoparticles. Schrand et al. [19] observed varying degrees of cytotoxicity from the same hydrocarbon-coated silver nanoparticles in different cell lines. Additionally, the different types of particles may explain some of the variabilities. For example, a suspension of water-soluble 10 nm silver nanoparticles [49] exhibited toxicity in the HepG2 cell line at a concentration of about 3.6 *μ*g mL^−1^ as opposed to 10 nm polyethylenimine-coated silver nanoparticles which caused toxicity in HepG2 cells at a concentration of 1 mg mL^−1^ [48]. One conclusion is that the particular particle type used in this study, 30–50 nm PVP-coated silver nanoparticles manufactured by NanoAmor (Houston, TX, USA), is relatively nontoxic to A549 cells at the ALI and only mildly toxic at higher doses in suspension. The size-dependent effects were not conclusive, as no toxic or inflammatory response was statistically significant compared to the control group. However, the data suggested that the 50 nm and 75 nm particles may be more toxic than the 100 nm particles despite having much lower mass doses than the larger particles. A recent study also found size-dependent results; suspensions of 5 nm PVP-coated silver nanoparticles were toxic at a concentration of 6.25 *μ*g mL^−1^ while 100 nm particles showed no toxicity even at the highest dose of 25 *μ*g mL^−1^ [50]. Further study is needed to confirm whether a size-dependent effect exists for silver nanoparticles.

### 4.2. ALI Exposure for Nanotoxicity Studies

Although the ALI exposure method is still in the early developmental stages, it is much less expensive and easier to perform than animal testing, while allowing for a controlled exposure with relatively well-characterized nanoparticle doses. While the ALI exposure is more difficult to carry out than conventional *in vitro* suspension exposures because of the added complexity of generating an aerosol and measuring particle deposition, it allows for an *in vitro* exposure to aerosolized particles in their native state and a more accurate determination of true cell dose. True cell dose for suspension exposures is impacted by particle aggregation in the culture medium and dependent upon particle transport through the medium to the cell surface. Following the analysis of Teeguarden et al. [51], assuming spherical monodisperse particles (~100 nm, from DLS measurement), we estimate that the majority of the particles have deposited on the cell surface in ~5 hr. This deposition time is comparable to the ALI dosing period of 2-3 hr. Considering the similar dosing periods, the greater response at the ALI compared to suspension is likely due to different particle physical/chemical characteristics rather than to differences in the dosing period. Another potential artifact of suspension exposures is that the particles may interfere with cellular response assays. We found that the silver nanoparticles prevented measurement of the LDH leakage in a dose-dependent fashion. Because of this interference, we suggest that future studies with silver nanoparticles investigate different measures of cellular response, such as the tightness of the monolayer, which will not be susceptible to such particle interferences.

A major limitation to the ALI approach is achieving adequate mass or number of particles depositing on the cells. The approach used in this study, like several others reported in the literature [52–55], relies on an electric field to enhance the deposition efficiency of charged particles onto the cell layer. It is possible that the charge on the particles may affect toxicity as gold nanoparticles with differently charged ligands have been shown to exhibit charge-dependent effects [56]. However, it is unlikely that the one or two extra positive charges on the silver nanoparticles will have a measurable impact on the particle toxicity. Another drawback to our ALI exposure method is the large degree of variation in the measures from replicate wells. A part of this variation is due to variation in dose, that is, the well-to-well deposition efficiencies. Our system achieved greater deposition efficiencies than systems relying on gravitational and diffusional deposition (7% [57]) as well as other systems employing electrostatic deposition (2% [52], 15–30% [53–55]). Well-to-well differences in deposition were larger than desired but similar to those of other systems, which had standard deviations as high as 30% [52]. We expect that much of the variation is due to uncertainties in the dose measurement rather than actual variations of the amount deposited. The fluorescein aerosol was assumed to be constant in time, so fluctuations of up to 10% in the aerosol concentration stemming from instabilities in the aerosol generator and uncertainty in the CPC measurement added to the uncertainty of the calculated deposition efficiency. An additional factor contributing to the large variation among cellular responses was the difficulty of culturing and exposing cells on the upside-down Transwell. Cells were not always plated uniformly because the cell suspension did not always spread evenly across the Transwell bottom. Evidence of this could be seen in well-to-well variations of the filtered air control that were in some cases larger than the variation seen in the deposition efficiencies. However, using the Transwells in the upside-down orientation was necessary to avoid losses of nanoparticles to the Transwell walls and achieve a measurable deposition of nanoparticles. We expect that a considerable amount of the variation could be reduced if a modified Transwell or alternative culturing methods could be developed.

A novel aspect of this work was the ability to restrict exposure to nanoparticles of a single diameter. Additionally, with the ALI we were able to determine the particle number dose for each condition and to compare the results using different dose metrics. Although surface area has frequently been used as a metric to explain particle effects [36], number dose has not been adequately investigated as a dose metric perhaps because of the difficulty of determining the number dose with conventional suspension exposures. We were only able to achieve low mass doses with our system and were unable to detect a significant cellular response with the rather innocuous silver nanoparticles. We expect that larger doses could be achieved with our exposure system by using a unipolar charger to improve the charging efficiencies of nanoparticles [58] and using a coarser size selection method as opposed to a DMA, which selects a very narrow size range of the aerosol. Additionally, a different aerosolization method capable of generating higher concentrations of monodisperse nanoparticles, such as electrospray, might be considered [59].

## 5. Conclusions

This research has shown the ALI dosing method to be effective at delivering microgram quantities of nanoparticles to the cell surface within a few hours. Additionally, the ALI approach can be used to expose cells to nanoparticles of a single diameter, albeit at low doses. The silver nanoparticles used in this study caused minimal cytotoxicity and only a mild inflammatory response. These results are consistent with the minimal response observed in rat inhalation exposures at lower concentrations [15]. Indications of a size-dependent response were observed but were not conclusive. The ALI method shows great promise for investigating the size dependence of nanoparticle toxicity and should be developed further because of its physiologically relevant exposure technique. Future methodological development should focus on increasing the concentrations of particles of a single diameter that can be delivered to the cell surface and reducing variability in the deposition efficiency.

## Figures and Tables

**Table 1:** Deposition efficiency (median and 25th and 75th percentiles) of fluorescein particles on the cell culture surface. Efficiencies are averaged over three replicate measurements and the three chamber inlets used for the cell exposures, except for 50 nm diameter particles, which were measured in four replicate experiments.

Diameter (nm)	Deposition efficiency (%) Median (25th, 75th)
50	38.2 (32.5, 63.1)
75	63.3 (53.1, 74.9)
100	63.5 (52.7, 75.5)

**Table 2:** Cellular response to nanoparticles dosed in suspension and at the ALI (median and 25th and 75th percentiles of three replicate wells for each condition, except where noted). Doses are presented per unit cell growth area, and responses are presented as percent control (ALI control is filtered air) to compare across several different experiments.

Material	Exposure	Dose (*μ*g cm^−2^)	MTT (% control) Median (25th, 75th)	LDH leakage (% control) Median (25th, 75th)	IL-8 (% control) Median (25th, 75th)
Silver	Suspension	2.6	94 (86, 97)	97 (95, 99)^a^	96 (94, 100)
		6.6	88 (83, 94)	95 (94, 98)^a^	98 (96, 102)
		13.2	80 (77, 85)*	92 (91, 96)^a^	112 (105, 122)
	ALI	0.7 (0.6, 0.7)	110 (66, 185)	96 (91, 265)^a^	136 (19, 389)

Nickel oxide	Suspension	2.6	93 (80, 97)	101 (99, 103)	—
		6.6	88 (76, 91)*	105 (102, 106)	105 (77, 117)
		13.2	83 (79, 86)*	107 (106, 109)*	87 (58, 89)
	ALI	2.1 (1.8, 2.2)	32 (14,76)	180 (160,324)*	15 (14, 44)*

*Statistically significant at a *P* value of 0.05. ^a^Values may be artificially low as silver nanoparticles were found to prevent the measurement of LDH protein.

**Table 3:** Number, surface area, and mass dose (median and 25th and 75th percentiles) of silver nanoparticles applied to cells as a function of diameter.

Diameter (nm)	Number (# × 10^6^ cm^−2^) Median (25th, 75th)	Surface Area (mm^2^ cm^−2^) Median (25th, 75th)	Mass (ng cm^−2^) Median (25th, 75th)
50	7.6 (4.0, 8.7)	0.06 (0.03, 0.07)	5 (3, 6)
75	5.5 (4.7, 6.5)	0.10 (0.08, 0.12)	13 (11, 15)
100	4.7 (3.9, 5.5)	0.15 (0.12, 0.17)	26 (22, 30)
